# Supplementary figures and images for: Influence of farmland confirmation on farmland abandonment in China
Source: PLoS One. 2023 May 4;18(5):e0285174. doi: 10.1371/journal.pone.0285174 (PMC10159111; doi:10.1371/journal.pone.0285174)

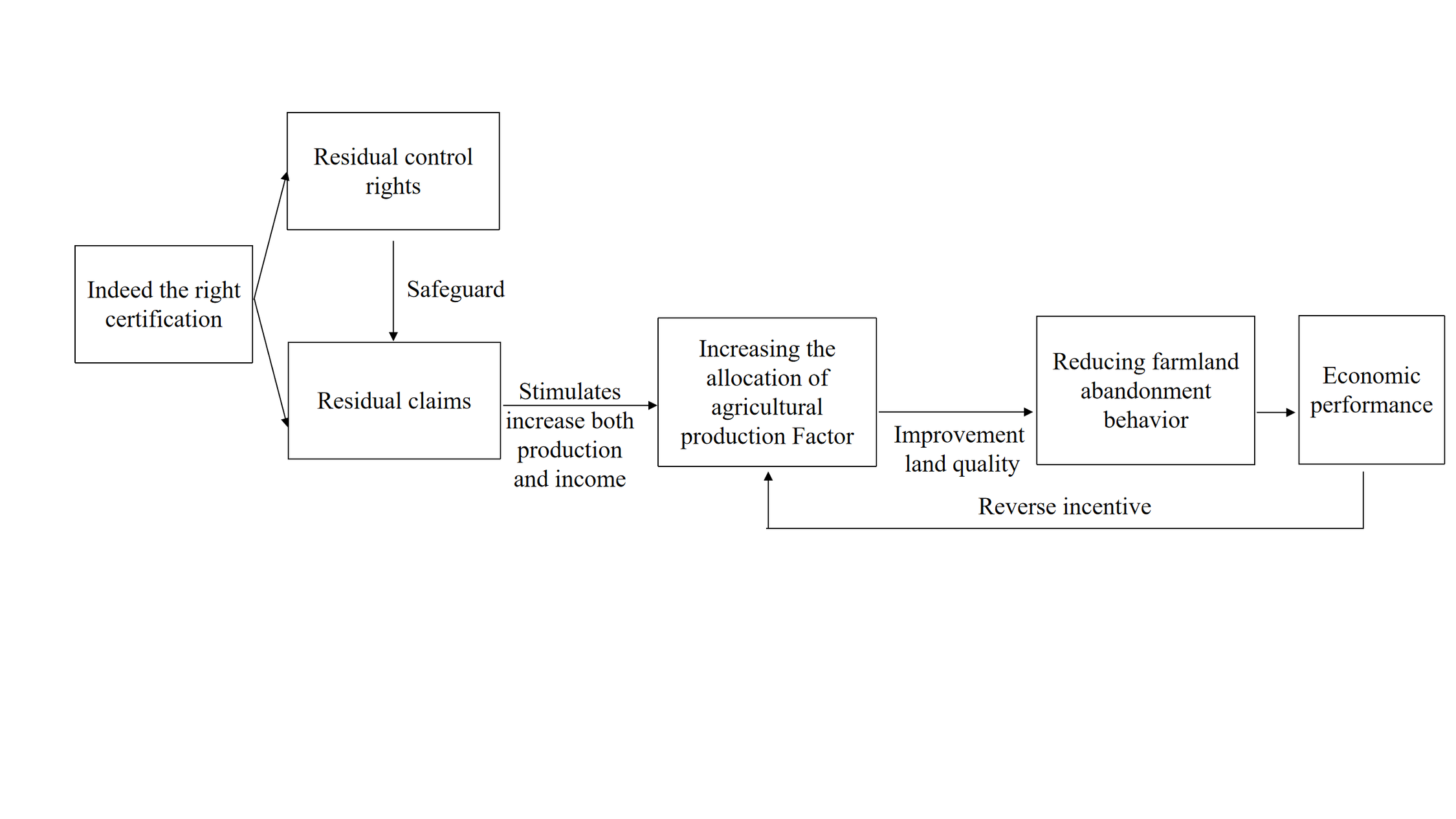

Supplement: S1 Fig — (TIF) [file pone.0285174.s001.tif]

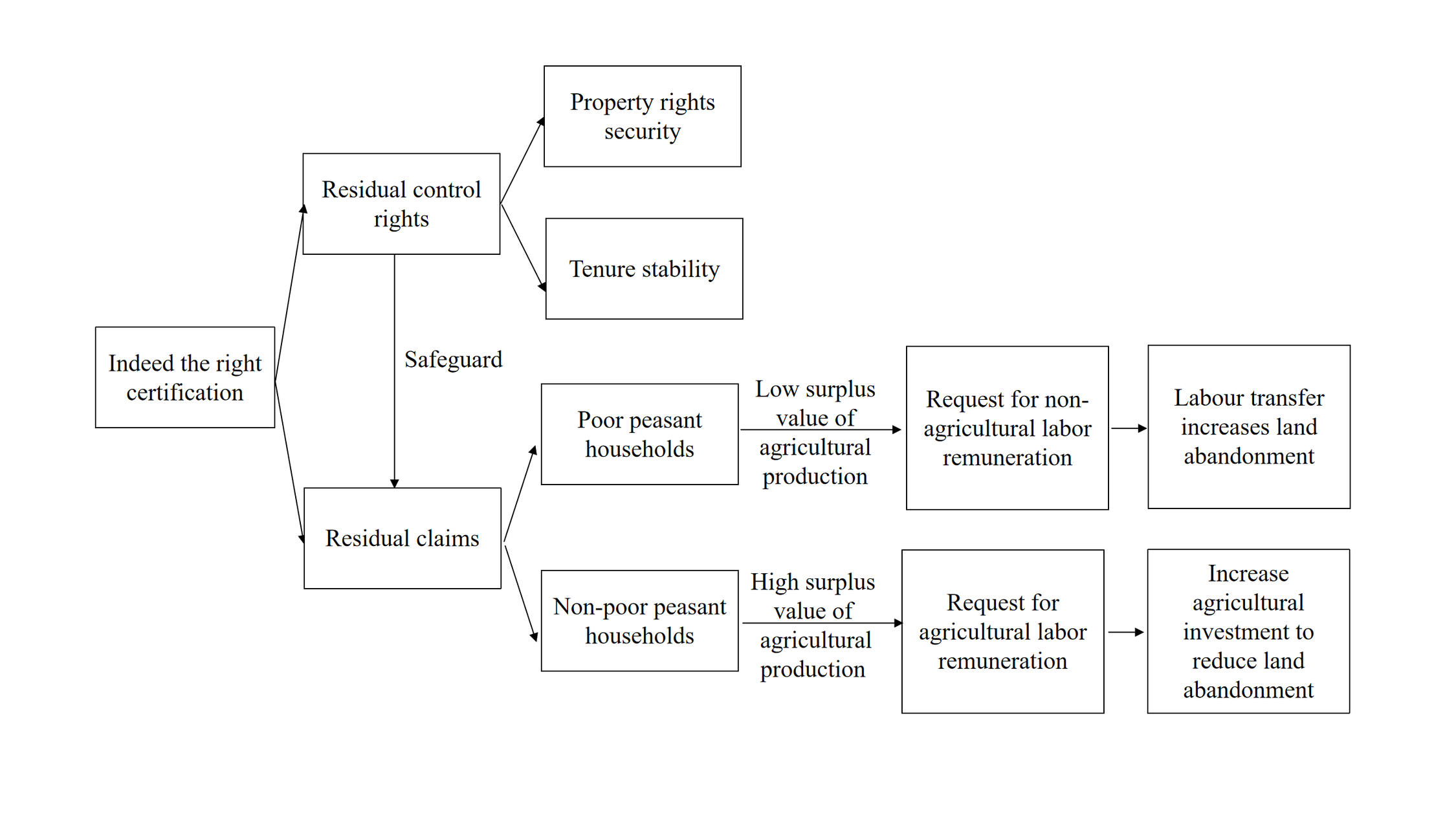

Supplement: S2 Fig — (TIF) [file pone.0285174.s002.tif]
