# Supplementary material for: Influence of farmland confirmation on farmland abandonment in China
Source: PLoS One. 2023 May 4;18(5):e0285174. doi: 10.1371/journal.pone.0285174 (PMC10159111; doi:10.1371/journal.pone.0285174)
Supplement: Attachment — Submitted filename: Response to Reviewers.docx [file pone.0285174.s005.docx]

**Detailed Response to Reviewers**

**Dear Editors, Dr. Essiagnon John-Philippe Alavo and Reviewers:**

Thank you for your letter and for the reviewers’ comments concerning our manuscript entitled "Influence of Farmland Confirmation on Farmland Abandonment in China" (ID: PONE-D-22-26169). Those comments are all valuable and very helpful for revising and improving our paper, as well as the important guiding significance to our researches. We have studied comments carefully and have made correction which we hope meet with approval. We upload two manuscript, one of which is a separate file labeled 'Revised Manuscript with Track Changes' and the other one is a separate file labeled 'Manuscript'. Revised portion are marked in red in the paper. The main corrections in the paper and the responds to the comments are as following:

**1. Your ethics statement should only appear in the Methods section of your manuscript. If your ethics statement is written in any section besides the Methods, please delete it from any other section.**

We have checked our intake and removed the ethics statement from other locations.

**2. Please ensure that you have specified (1) whether consent was informed and (2) what type you obtained (for instance, written or verbal, and if verbal, how it was documented and witnessed). If your study included minors, state whether you obtained consent from parents or guardians. If the need for consent was waived by the ethics committee, please include this information. Your ethics statement should only appear in the Methods section of your manuscript. If your ethics statement is written in any section besides the Methods, please delete it from any other section.**

Data Availability Statement: China Labor Force Dynamic Survey (CLDS) is a publicly available dataset. When applying for the right to use the data, the data user should provide true personal information to the Social Science Research Center of Sun Yat-sen University (hereinafter referred to as the “Data Provider”) and submit the completed “Basic Information of Data User Applicant Form” to the “Data Provider” via csyjzxsys@163.com, by fax or by mail to the “Data Provider”.

Ethics statement: Our research does not involve human participants.

**3. Please ensure that your manuscript meets PLOS ONE's style requirements, including those for file naming.**

We carefully read the PLOS ONE style templates and edit our manuscript according to the guidelines for main body, affiliations and file naming to ensure our revised manuscript meets PLOS ONE's style requirements.

**4.Thank you for stating in your Funding Statement:Please provide an amended statement that declares *all* the funding or sources of support (whether external or internal to your organization) received during this study, as detailed online in our guide for authors at http://journals.plos.org/plosone/s/submit-now. Please also include the statement “There was no additional external funding received for this study.” in your updated Funding Statement.**

We carefully read a lot of articles from PLOS ONE and modified our funding statement.

“**Funding:** This work was supported by National Social Science Foundation of China (No.20&ZD170); and the Major projects of National Social Science Found of China (21&ZD090)；and Academic year 2019-2020 Doctoral Dissertation Scholarship of Tsinghua Rural Studies (No.201901).The funders had no role in study design, data collection and analysis, decision to publish, or preparation of the manuscript.”

**5. Thank you for stating the following in your Competing Interests section: Please complete your Competing Interests on the online submission form to state any Competing Interests. If you have no competing interests, please state "The authors have declared that no competing interests exist.", as detailed online in our guide for authors at http://journals.plos.org/plosone/s/submit-now**

**This information should be included in your cover letter; we will change the online submission form on your behalf.**

We carefully read a lot of articles from PLOS ONE and modified our competing interests.

“**Competing interests:** The authors have declared that no competing interests exist.”

**6. PLOS requires an ORCID iD for the corresponding author in Editorial Manager on papers submitted after December 6th, 2016. Please ensure that you have an ORCID iD and that it is validated in Editorial Manager.**

We have validated the ORCID ID in Editorial Manager.

**7. Thank you for stating the following in the Acknowledgments Section of your manuscript:We note that you have provided additional information within the Acknowledgements Section that is not currently declared in your Funding Statement. Please note that funding information should not appear in the Acknowledgments section or other areas of your manuscript. We will only publish funding information present in the Funding Statement section of the online submission form.**

We have removed the funding-related text from the manuscript and update our Funding Statement in our cover letter.

“**Funding:** This work was supported by National Social Science Foundation of China (No.20&ZD170); and the Major projects of National Social Science Found of China (21&ZD090)；and Academic year 2019-2020 Doctoral Dissertation Scholarship of Tsinghua Rural Studies (No.201901).The funders had no role in study design, data collection and analysis, decision to publish, or preparation of the manuscript.”

**8. Please ensure that you refer to Figure 2 in your text as, if accepted, production will need this reference to link the reader to the figure.**

We are sorry for the omission of Figure 2 in the article and we have clarified the placement of in our text.

**9. We note that several of your files are duplicated on your submission. Please remove any unnecessary or old files from your revision, and make sure that only those relevant to the current version of the manuscript are included.**

We are sorry for this mistake and correct it in our submission.

**10. Please ensure that you have specified (1) whether consent was informed and (2) what type you obtained (for instance, written or verbal, and if verbal, how it was documented and witnessed). If your study included minors, state whether you obtained consent from parents or guardians. If the need for consent was waived by the ethics committee, please include this information.**

Informed consent: We used the data of the China Labor-Force Dynamic Survey (CLDS). The CLDS was granted ethical approval from the Research Ethics Committees of the Social Science Research Center of Sun Yat-sen University. All subjects signed written informed consent before the interview.

**11. Please ensure that the title on the details page and on the title page of the manuscript are identical.**

We are ensure that the title on the details page and on the title page of the manuscript are identical.

**12. Please upload a copy of all Figures which you refer to in your text. Or if the figure is no longer to be included as part of the submission please remove all reference to it within the text.**

We have upload all Figures, Tables, and Files mentioned in our text as Supporting information files in Editorial Manager.

**13. We note your Data Availability Statement as follows:**

We have upload our underlying data as Supporting information files in Editorial Manager and update our Data Availability Statement.

**Data Availability Statement:** China Labor-Force Dynamic Survey (CLDS) is a publicly available dataset. When applying for the right to use the data, the data user should provide true personal information to the Social Science Research Center of Sun Yat-sen University (hereinafter referred to as the "Data Provider") and submit the completed "Basic Information of Data User Applicant Form" to the "Data Provider" via csyjzxsys@163.com, by fax or by mail to the "Data Provider".

**Response to Reviewer #1:**

Thank you for your careful and thoughtful examination of our paper. Incorporating your comments, as well as those of the Editor, and the other referee, resulted in a greatly improved manuscript. We address each of your comments below.

**1. The explained variable uses "whether farmers give up farmland, only using yes or no, but some people have not completely given up farmland. Whether we are considering whether we can use several options to distinguish intensity, which can more accurately reflect farmers' attitude to farmland. Even if there is a robust test, the measurement of this variable is not appropriate.**

In our explained variable "whether farmers give up farmland", the use of no means that the farmland has been abandoned. In order to differentiate the intensity, we use the specific number of farmers abandoned (Waste area of cultivated land and Irrigated land abandoned) in our robust test to differentiate the intensity and thus verify the robustness of our study.

**2. There is no econometric model in the paper.**

We have added to the econometric model in the paper.

To validate the influence of Farmland Confirmation on Farmland Abandonment, we consider the following model:

*Y=β_0_+β_1_Farmland confirmation+β_2_Control variables+ɛ*	(1)

In Eq. (1), *Y* is the explained variable, and *Farmland confirmation* is the Explanatory variable. *Control variables* is a set of control variables and *ɛ* denotes the error term.

**Response to Dr.Essiagnon John-Philippe Alavo**

Thank you for your careful and thoughtful examination of our paper. Incorporating your comments, as well as those of the Editor, and the other referee, resulted in a greatly improved manuscript. We address each of your comments below.

Very good topic and timely to the moment and well presented. However, there are minor things to consider for the paper to be ready for publication. **Please kindly read the authors guide and appropriately correct the minor grammatical errors in the paper in order to make it more suitable to the journal. And also consider sections titles and numbering. Also, the authors need to present the analytical framework in detail supported by literature, define and describe the variables used in the study and explain the model employed.**

Firstly, we carefully read the authors guide from PLOS ONE and edit our manuscript according to the guidelines for main body to ensure our revised manuscript meets PLOS ONE's style requirements. Secondly，we read our paper carefully and corrected the minor grammatical errors in the paper. Thirdly, we carefully angled and numbered the titles of our paper. Fourthly，we present the analytical framework in detail supported by literature and provide Figure 1 and Figure 2 to help the reader understand the idea of our study. Finally, we define and describe the variables used in the study and explain the model employed.

**Finally, we would like to express our gratitude to the editor and all the reviewers for the extremely helpful comments and for your guidance in the revision. We tried our best to improve the manuscript and made some changes in the manuscript. These changes will not influence the content and framework of the paper. We hope that our efforts have succeeded in allaying your concerns. We look forward to learning about your decision. And we express our thanks again to the referee for his time and efforts in reviewing our paper. Any remaining errors are our own.**

**Thank you and best regards.**
